# The overlooked ZIF: reviving [Cu(imidazolato)_2_] from 1960s literature

**DOI:** 10.1107/S2052252526008912

**Published:** 2026-10-20

**Authors:** Cara Bosman, Shane G. Telfer

**Affiliations:** a MacDiarmid Institute for Advanced Materials and Nanotechnology, School of Food Technology and Natural Sciences Massey University Palmerston North4410 New Zealand; Stockholm University, Sweden

**Keywords:** zeolitic imidazolate frameworks, metal–organic frameworks, gas adsorption, [Cu(im)_2_], polymorphs, isotherms

## Abstract

Metal–organic frameworks (MOFs), and in particular zeolitic imidazolate frameworks (ZIFs), are amongst the most well studied materials in modern chemistry. MOFs emerged as an explicit subfield of chemistry in the late 1980s and 1990s. However, much foundational work was accomplished in earlier decades. Here, we detail the structural and gas-adsorption characterization of [Cu(im)_2_] (im = imidazolate), a ZIF that was widely reported in the early 1960s. This compound exists as several polymorphs. We re-prepared the historic blue polymorph [Cu(im)_2_]-B, which has a sodalite topology, confirmed its identity by single-crystal X-ray diffraction, and measured its gas-adsorption isotherms. We conclude that the true origin of ZIFs lies with a copper(II) framework discovered more than 60 years ago that has been overlooked by recent literature.

## Abstract 2

This study investigates [Cu(im)_2_], a zeolitic imidazolate framework (ZIF) originally discovered in the 1960s but widely overlooked since. We characterize its B polymorph with the sodalite topology. By measuring its gas-adsorption isotherms to establish its permanent porosity, we demonstrate that the origins of modern ZIFs lie much earlier than previously recognized.

## 1. Introduction

The 2025 Nobel Prize in Chemistry, awarded to Susumu Kitagawa, Richard Robson and Omar Yaghi ‘*for the development of metal–organic frameworks*’, marks the spectacular rise of a field that, only three decades ago, did not yet have a name. The award acknowledges the discoveries made by the laureates’ contributions in taking ‘*the steps from analysis to prediction and then to controlled synthesis*’ (Hoskins & Robson, 1990 ▸; Kondo *et al.*, 1997 ▸; Li *et al.*, 1999 ▸; The Royal Swedish Academy of Sciences, 2025 ▸). The Nobel committee’s timeline is necessarily a recent one, but the chemistry it honours has historical roots. The scientific foundations were laid around a century ago by Werner (1893 ▸), Hofmann & Küspert (1897 ▸) and Keggin & Miles (1936 ▸) in the form of the structure and bonding of coordination compounds and the use of coordination chemistry to induce porosity in extended materials. The term ‘coordination polymer’ can be traced back to 1916 (Batten & Champness, 2017 ▸). What the rapid rise of metal–organic framework (MOF) literature can obscure, however, is that several of the simplest MOF materials had already been synthesized, characterized, and published as crystalline solids long before the term MOF existed. This includes the apparent report of a beryllium analog of MOF-5 in 1958 (Marvel & Martin, 1958 ▸) and the recognition of the ‘zeolitic’ properties of [Ca(squarate)] (Robl & Weiss, 1987 ▸). The permanent porosity of both of these materials was established much more recently (Porter *et al.*, 2009 ▸; Li *et al.*, 2019 ▸).

Within the MOF family, the class of metal azolate frameworks occupies a privileged position. They are built from metal ions coordinated to imidazolate (im^−^), pyrazolate, triazolate and tetrazolate anions (Zhang *et al.*, 2012 ▸). ZIFs, in particular, combine chemical robustness with the topological richness of zeolites. A survey of metal azolate frameworks by Zhang & Chen (2006 ▸) drew together much of this chemistry, and the ZIF terminology was introduced later that year (Park *et al.*, 2006 ▸). From a historical context, a dense [Zn(im)_2_] compound with tetrahedral zinc(II) centres and the zni topology was reported in 1980 (Lehnert & Seel, 1980 ▸). The first zinc(II) compound to be recognized as having a zeolitic topology and accessible porosity was [Zn(benzimidazolate)_4_] (Huang *et al.*, 2003 ▸), which was followed by a report of the structure and gas-adsorption isotherm of [Zn(2-methylimidazolate)_2_], later known as ZIF-8 (Huang *et al.*, 2006 ▸). The family has since expanded to include several dozen distinct structures (Zheng *et al.*, 2023 ▸), and ZIF-8 has become one of the most widely investigated porous materials. The ZIF literature has concentrated on Zn(II), and to a lesser extent Co(II), analogues whose strict tetrahedral coordination geometry is naturally compatible with zeolitic topologies. While Cu(II) usually exhibits a preference for square planarity, its coordination geometry can distort to a sufficient degree to allow zeolitic topologies to assemble.

The case of [Cu(im)_2_] is especially striking: this compound was widely reported in the chemical literature in the early 1960s. In a number of cases the original authors anticipated, on chemical grounds alone, that their products were polymeric coordination networks. However, the language of ‘frameworks’ and ‘porosity’ was not part of standard lexicon and the gas-adsorption instruments required to experimentally demonstrate gas uptake were not standard laboratory equipment. A historical reassessment in honour of the 2025 Nobel Prize is a natural moment to revisit this material. We would like to draw attention to the genesis of ZIFs, which seemingly lies with a Cu(II)–imidazolate framework first prepared much earlier than any Zn(II) material. This deserves to be celebrated as a scientific landmark.

Jarvis and Wells, working at the Imperial Chemical Industries, UK, reported a single-crystal X-ray structure of ‘copper(II) imidazole’ at the Sixth International Congress of the IUCr in 1960 (Jarvis & Wells, 1960 ▸). Their abstract, published in *Acta Crystallographica*, mentions a monoclinic unit cell (*a* = 11.75, *b* = 14.07, *c* = 8.77 Å, β = 97.2°), space group *I*2/*c*, with the structure refined to *R* = 15.5%. The connectivity is described as ‘*a three-dimensional network of Cu atoms linked by imidazolate molecules*’ with two crystallographically distinct Cu(II) sites. Jarvis and Wells qualify the report explicitly as describing ‘*one polymorph*’ of the compound, alerting readers to the polymorphism inherent to [Cu(im)_2_]. This polymorphism had been hinted at in earlier work focusing on the solution chemistry of imidazole and copper(II) (Edsall *et al.*, 1954 ▸). The landscape of polymorphs would be later mapped out in detail (*see infra*) and the J polymorph ascribed to Jarvis and Wells’ structure {[Cu(im)_2_]-J}. To our knowledge, the 1960 report is the first explicit structural description of a three-dimensional [Cu(im)_2_] framework. While a full paper was promised this never seems to have materialized; fortunately the structure did re-enter the open literature seven years later in the form of a table of atomic coordinates communicated by Wells to H. C. Freeman (Freeman, 1967 ▸). Freeman, whose motivation was strictly bioinorganic, presciently assigned the structure the topological label ‘PtS-type’ on the basis of Wells’ own three-dimensional-net classification. These are the same descriptors that the MOF community would adopt decades later. The link between simple inorganic structures and extended materials was the inspiration behind Robson’s formative work in the area of coordination polymers (Robson, 2000 ▸).

**Figure 1:**
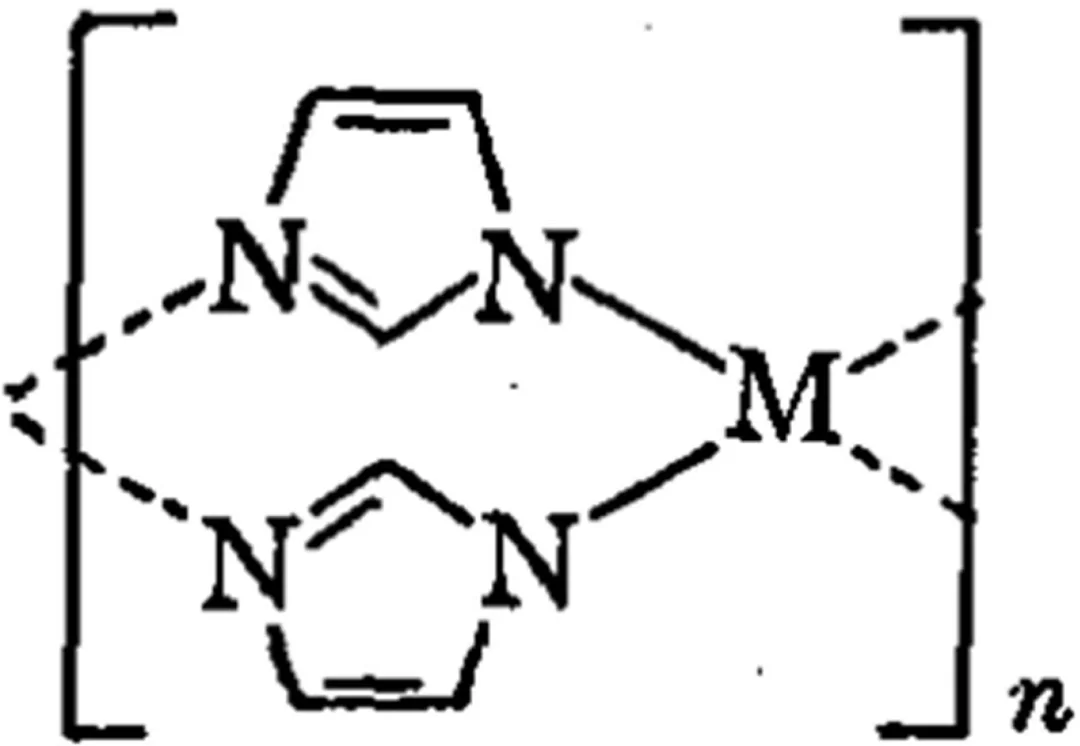
The extended polymeric chain motif of crystalline metal–imidazolate materials proposed by Brown & Aftergut (1964 ▸). Reproduced with permission. Copyright (1964) John Wiley & Sons, Inc.

A different niche of the 1960s literature contains another independent report of [Cu(im)_2_]. In 1964, Brown and Aftergut, working at the General Electric Advanced Technology Laboratories, USA, prepared a series of ‘bis(imidazolato)-metal polymers’ by reaction of imidazole with the sulfates of Cu(II), Zn(II) and Co(II) in aqueous sodium bicarbonate (Brown & Aftergut, 1964 ▸). A blue precipitate from the copper reaction was assigned the formula [Cu(im)_2_] and found to be crystalline with sharp powder X-ray diffraction (PXRD) peaks coinciding with what would later be referred to as the B polymorph. Brown and Aftergut explicitly proposed an extended bridging-imidazolate framework, drawing the polymeric motif implying a 1D chain (Fig. 1 ▸). Their reference list contains no mention of the Jarvis–Wells work; in the days of slow communications and trips to the library to consult references it is unsurprising that the connection between the two early reports of crystalline [Cu(im)_2_] does not appear to have been made. Furthermore, the Brown & Aftergut paper appeared in the *Journal of Polymer Science*, likely an outlet infrequently consulted by inorganic chemists. Its disappointing electrical resistivities (the property they had hoped to optimize) effectively closed the file on their work.

Around the same time, Bauman Jr and Wang at the University of Missouri, USA, reported the isolation of a reddish-purple [Cu(im)_2_] phase from Cu(NO_3_)_2_ and aqueous NaOH (Bauman & Wang, 1964 ▸). This is plausibly the ‘pink P’ polymorph. Bauman and Wang also argued for an extended structure: ‘*the ionization of the pyrrole hydrogen changes the monodentate imidazole molecule into the bidentate imidazolate anion. This in turn raises the possibility of the formation of a polymeric compound.*’ Tellingly, they cited unpublished single-crystal X-ray work by C.-I. Brändén in Uppsala showing that [Zn(im)_2_] is ‘*an infinite polymer in which the metal ion is tetrahedrally surrounded by four ligand molecules*’. As an aside, further details of the structure of this [Zn(im)_2_] do not appear to have been published, so the tantalizing possibility that this was a porous ZIF remains. A further report on [Cu(im)_2_] came from Inoue *et al.* (1965 ▸) at Osaka University, Japan, who in 1965 reported the magnetic susceptibility of [Cu(im)_2_]-B, citing Brown and Aftergut for the synthetic method, and inferred it ‘*should be polynuclear*’ with the metal centres bridged through the azolate nitrogen atoms. Collectively, the Jarvis & Wells (1960 ▸), Brown & Aftergut (1964 ▸), Bauman & Wang (1964 ▸) and Inoue *et al.* (1965 ▸) reports constitute four independent lines of evidence that describe a common result: [Cu(im)_2_] forms a suite of polymorphic coordination polymers that can be crystalline.

Four decades later, an independent re-investigation of the polymorphs of [Cu(im)_2_] by Masciocchi and co-workers collated and extended our understanding of these materials (Masciocchi *et al.*, 2001 ▸). Five polymorphs were discussed: blue J, blue B, green G, olive O and pink P. They solved the structures of all but the J and P forms by PXRD. The B polymorph, identical to Brown and Aftergut’s, crystallizes in the *R*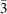 space group with visible channels; the ‘olive O’ form (space group *C*2/*c*) adopts the mog topology with internal cavities; and the ‘green G’ form (space group *Ccca*) consists of two-dimensional slabs.

The sod topology of [Cu(im)_2_]-B is shared by several prom­inent ZIFs, including ZIF-8 {[Zn(2-methylimidazolate)_2_]} and its benzimidazolate congener, ZIF-7 (Park *et al.*, 2006 ▸). Strikingly, although at least ten distinct [Zn(im)_2_] phases have been reported, none have the sod topology (Zheng *et al.*, 2023 ▸). Consequently, [Cu(im)_2_]-B is the structurally simplest member of the ZIF family to exhibit this architecture. Also, among the known polymorphs, only this one features pore dimensions suitable for the adsorption of guest molecules (Table 1 ▸), as demonstrated by a low-temperature adsorption isotherm of CO_2_ (Xue *et al.*, 2024 ▸). Here, we revisit this material more than six decades after its initial synthesis to map out its microporous properties. In doing so, we bridge the gap between the historical origins of [Cu(im)_2_] in the early 1960s, its structural rediscovery a generation later, and its rightful place in MOF history.

## 2. Experimental

### 2.1. Hydrothermal synthesis of [Cu(im)_2_]-B

Imidazole (130 mg, 1.9 mmol, 2 eq.), NaHCO_3_ (304 mg, 3.6 mmol, 3.8 eq.), CuSO_4_·5H_2_O (240 mg, 0.96 mmol, 1 eq.) and water (6 ml) were combined in a 20 ml glass vial. The mixture was briefly sonicated, and the vial was sealed and heated in an oven at 85°C overnight to give purple–blue needle-like crystals. The crystals were isolated by vacuum filtration while the solution was still warm, then washed with water and acetone. Yield: 113 mg (59%).

### 2.2. Large-scale synthesis of [Cu(im)_2_]-B

Imidazole (15.1 g, 0.22 mol, 2 eq.) and NaHCO_3_ (37.6 g, 0.45 mol, 4 eq.) were dissolved in water (300 ml) in a round bottom flask, with stirring while heating in an oil bath maintained at 100°C. To this mixture, a solution of CuSO_4_·5H_2_O (27.8 g, 0.11 mol, 1 eq.) dissolved in water (200 ml) was added slowly. The resulting suspension was heated at 100°C for 18 h. The solid product was collected by vacuum filtration, washed thoroughly with water, followed by washing thoroughly with acetone, and dried at 120°C. This afforded the product a dark blue powder. Yield: 20.2 g (90%).

### 2.3. Pelletization of [Cu(im)_2_]-B

A Caleva Multi-Lab Classic system paired with a syringe pump was used. [Cu(im)_2_]-B powder (14.55 g) was placed in the Caleva chamber and an aqueous polyvinyl alcohol (PVA) solution (45 mg ml^−1^, 12 ml) was added over 10 min while mixing at 50 r min^−1^. The mixture was then blended for an additional 10 min at 100 r min^−1^ to form a homogeneous dough. The dough was extruded through a 2 mm die, and the initial extrudates were collected and extruded a second time. The resulting cylindrical noodles were dried in an oven at 120°C then cut into pellets with lengths around 3–4 mm.

### 2.4. X-ray diffraction

Single-crystal data, data collection and structure refinement details are summarized in Table 2 ▸. H atoms were placed in geometrically calculated positions and refined with a riding model with *U*_iso_(H) = 1.2*U*_eq_(C) (where *U*_iso_ is the isotropic displacement parameter of a specific hydrogen atom and *U*_eq_ is the equivalent isotropic displacement parameter of the carbon atom to which it is bonded). Powder diffraction data were measured on the same instrument, hence the slight peak broadening. The data were measured by rotating at phi 360° min^−1^ over 1 min and were processed into 1D diffractograms using *APEX3* and *Diffrac.Eva*. Predicted PXRD patterns were generated from CIFs using *Mercury*.

### 2.5. Gas-adsorption analysis

For gas-adsorption measurements, samples were exchanged with acetone then activated under high vacuum at 100°C overnight. Isotherms were measured on a Quantachrome Autosorb iQ2 instrument using ultra-high purity gases.

### 2.6. Gas-breakthrough analysis

For breakthrough measurements, 10.98 g of [Cu(im)_2_]-B pellets were activated under vacuum at 100°C in a stainless steel column (16 × 98 mm), then gas-breakthrough data were recorded at 293 K using a previously reported setup (Qazvini *et al.*, 2019 ▸) with a CO_2_/N_2_ (15/85) gas mixture with a total flow rate of 10 ml(STP) min^−1^ (STP represents being corrected to standard temperature and pressure conditions) and a pressure of 1 bar.

## 3. Results and discussion

**Figure 2:**
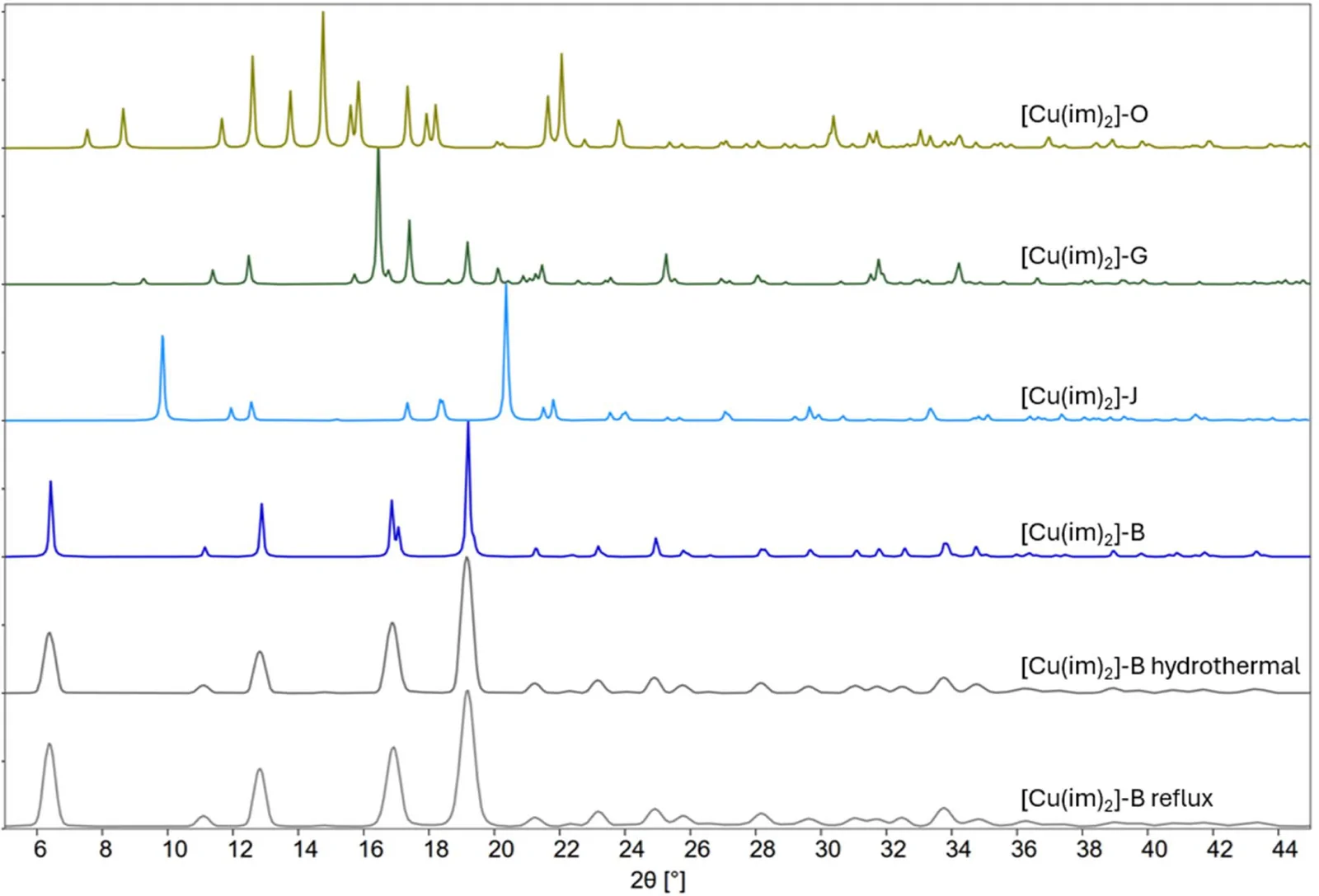
PXRD patterns of the O, G, J and B polymorphs of [Cu(im)_2_] calculated from coordinates obtained from the literature alongside the experimental patterns of [Cu(im)_2_]-B obtained by the hydrothermal and large-scale reflux synthetic methods. The PXRD patterns of as-synthesized and activated [Cu(im)_2_]-B are identical.

We found that while Brown and Aftergut’s synthetic method (Brown & Aftergut, 1964 ▸) delivers [Cu(im)_2_]-B in reasonable yield, it is quite protracted, taking three days. To shorten the reaction time, we took two different approaches. First, a small-scale hydrothermal synthesis route delivered purple–blue needle-like single crystals overnight in good yield. These crystals diffracted well and we determined the structure by single-crystal X-ray diffraction (Table 2 ▸). The structure matched that previously determined by PXRD (Masciocchi *et al.*, 2001 ▸). Second, the reaction was scaled up by simple refluxing in water and a reaction time of 18 h, which yielded a microcrystalline purple–blue solid. The PXRD patterns of the two products are identical and match the pattern simulated from the crystal structure (Fig. 2 ▸). The *d* spacings reported by Brown and Aftergut at 6.90, 5.10 and 4.60 Å coincide with the intense reflections observed at 2θ = 12.8° (220), 16.8° (101) and 19.2° (12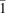), respectively.

**Figure 3:**
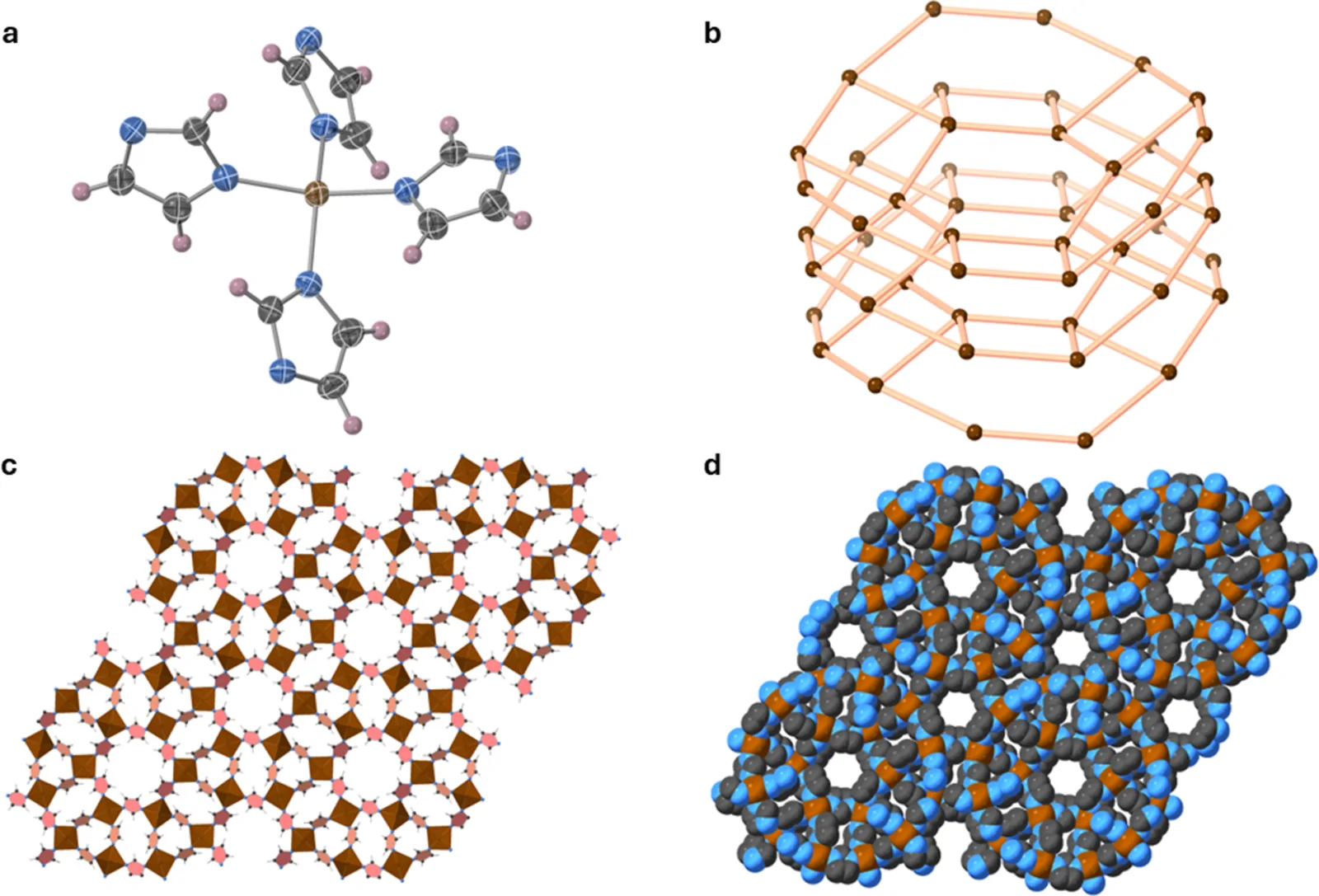
The structure of [Cu(im)_2_]-B as deduced by single-crystal X-ray diffraction: (*a*) the arrangement of four imidizolato ligands around the copper(II) centre with ellipsoids shown at the 50% level, (*b*) the underlying sodalite topology, (*c*) a polyhedral view down the crystallographic *c* axis, and (*d*) the 1D channels viewed down the crystallographic *c* axis in space-filling mode (H atoms omitted for clarity). Colours: Cu = brown, C = dark grey, N = blue, H = pink, filled imidazolate rings = peach.

**Figure 4:**
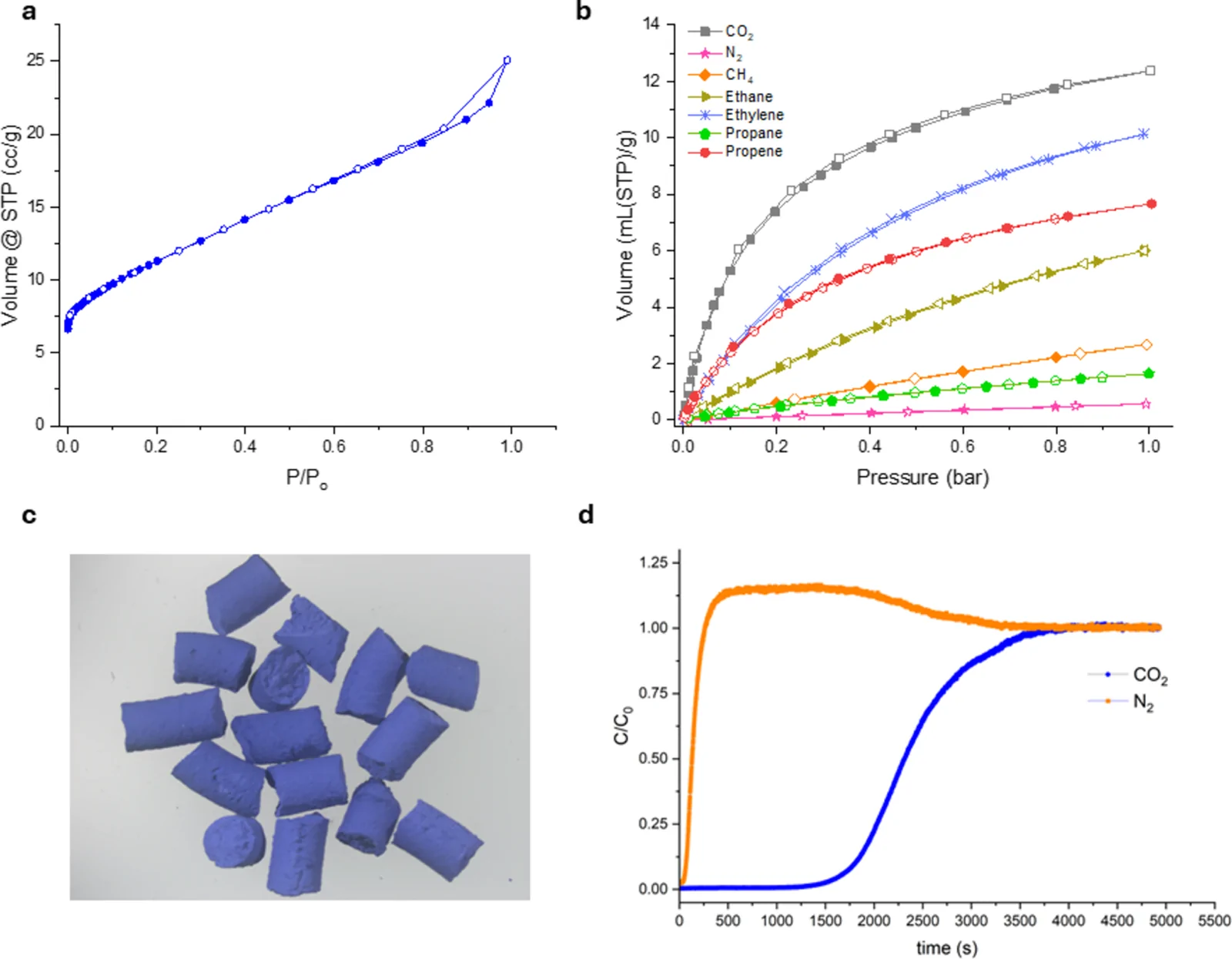
Gas-adsorption isotherms of [Cu(im)_2_]-B: (*a*) N_2_ uptake at 77 K and (*b*) various gases at 293 K, closed symbols = adsorption while open symbols = desorption. (*c*) Optical micrograph of the [Cu(im)_2_]-B pellets, which measure 2 mm in diameter and ∼3–4 mm in length. (*d*) Gas-breakthrough measurements using a column packed with pelletized [Cu(im)_2_]-B and a CO_2_/N_2_ (15/85) gas mixture [total flow rate = 10 ml(STP) min^−1^].

The structure of [Cu(im)_2_]-B, deduced here by single-crystal X-ray diffraction, shows a flattened coordination geometry around the copper(II) ion, which lies between tetrahedral and square planar with N–Cu–N bond angles ranging between 92.5° and 154.1° [Fig. 3 ▸(*a*)]. This distortion from a square-planar arrangement enables the imidazolate ligands to bridge the copper(II) nodes and generate the sodalite topology [Fig. 3 ▸(*b*)]. Space-filling models highlight one-dimensional channels running parallel to the crystallographic *c* axis, featuring a pore-limiting diameter (PLD) of ∼3.5 Å and a maximum pore diameter of 4.0 Å. Based on these structural features, the framework is expected to exhibit porosity toward guest molecules, and this has been established experimentally by a CO_2_ isotherm at 195 K (Xue *et al.*, 2024 ▸). Following activation at 100°C under vacuum to remove occluded water, we found [Cu(im)_2_]-B to exhibit a type I isotherm for N_2_ adsorption at 77 K and various other gases at 293 K (Fig. 4 ▸). The surface area derived from its low-temperature N_2_ isotherm is 39.7 m^2^ g^−1^, and its N_2_-accessible pore volume is estimated to be 0.030 cm^3^ g^−1^. This pore volume compares well with the value of 0.04 cm^3^ g^−1^ reported on the basis of a CO_2_ isotherm at 195 K (Xue *et al.*, 2024 ▸). At 293 K, we found [Cu(im)_2_]-B to adsorb a range of gases. It has a strong affinity for CO_2_, reaching an uptake of 12.4 ml(STP) g^−1^ at 1 bar, which contrasts with its low affinity for N_2_ [0.57 ml(STP) g^−1^ at 1 bar]. The CO_2_/N_2_ uptake ratio is 21.7. Additionally, the material discriminates between olefins and paraffins at 1 bar, yielding ethylene/ethane and propene/propane uptake ratios of 1.7 and 4.7, respectively.

These promising selectivities prompted us to conduct dynamic breakthrough experiments to evaluate its gas-separation performance when subject to a CO_2_/N_2_ (15/85) flow. [Cu(im)_2_]-B was pelletized by blending into a composite with PVA [Fig. 4 ▸(*c*)]. The Brunauer–Emmett–Teller (BET) surface area of the [Cu(im)_2_]-B pellets was measured by an N_2_ isotherm at 77 K to be 37.9 m^2^ g^−1^, showing that pelletization did not compromise its gas-adsorption capacity. Upon introducing the gas mixture into a packed bed of [Cu(im)_2_]-B pellets, a clean separation was achieved [Fig. 4 ▸(*d*)]. The weakly adsorbing N_2_ broke through the column almost immediately, whereas CO_2_ was retained for a significant period before breaking through. The equilibrium CO_2_ uptake obtained from the breakthrough experiment [5.42 ml(STP) g^−1^] can be compared with the CO_2_ uptake seen in the single-component CO_2_ isotherm of the [Cu(im)_2_]-B pellets at the equivalent partial pressure [5.87 ml(STP) g^−1^]. The small drop in capacity is due to a minor amount of competitive adsorption of N_2_ and non-isothermal effects. The reasonably sharp breakthrough profiles indicate low mass-transfer resistances, which is consistent with the rapid adsorption kinetics noted during the isotherm measurements.

## 4. Conclusions

[Cu(im)_2_] is both a chemically rich and historically significant material. The compound was first characterized structurally in 1960 by Jarvis and Wells, who solved the single-crystal X-ray structure of the J polymorph and recognized it as a three-dimensional coordination network geometry around the copper(II) ion being distorted from square planarity (Jarvis & Wells, 1960 ▸). Several years later, Freeman published Wells’ atomic coordinates and assigned the pts topology (Freeman, 1967 ▸). The B polymorph was reported in 1964 by Brown and Aftergut, who established a synthetic method, observed its crystallinity, and explicitly proposed an extended polymeric framework (Brown & Aftergut, 1964 ▸). Four decades later, Masciocchi and co-workers identified its sodalite topology and the channels in its crystal structure (Masciocchi *et al.*, 2001 ▸), which were later shown to accommodate CO_2_ at low temperature (Xue *et al.*, 2024 ▸). Subsequently, the topologically equivalency of [Cu(im)_2_] to the newly discovered ZIF-8 was established (Huang *et al.*, 2006 ▸). Now, by reproducing and improving Brown and Aftergut’s synthetic procedure, we have confirmed that the material they produced is capable of reversibly adsorbing and separating a range of gases.

[Cu(im)_2_]-B is a permanently microporous polymeric coordination network that predates the first reported Zn(II) imidazolate frameworks by four decades. In this light, it deserves recognition as the first ZIF published in the chemical literature. MOF chemistry has historical roots that are deeper than its modern prominence may suggest; other unrecognized materials may yet be hiding in obscure places.

## Supplementary Material

Crystal structure: contains datablock(s) I. DOI: 10.1107/S2052252526008912/kin5001sup1.cif

CCDC reference: 2583741

## Figures and Tables

**Table 1:** A summary of the reported polymorphs of [Cu(im)_2_]

Polymorph	First reported	Space group	Topology	Porosity†	
Blue, J	Jarvis & Wells (1960 ▸)	*I*2/*c*	pts	PLD = 1.9 Å, *V* = 0.002 cm^3^ g^−1^	
Blue, B	Brown & Aftergut (1964 ▸)	*R* 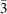	sod	PLD = 3.5 Å, *V* = 0.032 cm^3^ g^−1^	
Green, G	Inoue *et al.* (1965 ▸)	*Ccca*	2D	PLD = 1.2 Å, *V* = 0.008 cm^3^ g^−1^	
Olive, O	Masciocchi *et al.* (2001 ▸)	*C*2/*c*	mog	PLD = 1.9 Å, *V* = 0.073 cm^3^ g^−1^	
Pink, P	Bauman & Wang (1964 ▸)	Unknown	Unknown	Unknown	

† As determined by *PoreBlazer* (Sarkisov *et al.*, 2020 ▸). PLD = Pore-limiting diameter. *V* = Network-accessible helium volume.

**Table 2:** Single-crystal X-ray diffraction experimental details

Crystal data	
CCDC deposition number	2583741
Chemical formula	C_18_H_18_Cu_3_N_12_
*M*_r_	593.06
Crystal system, space group	Trigonal, *R* 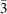
Temperature (K)	293
*a*, *b*, *c* (Å)	27.5070 (13), 27.5070 (13), 5.3922 (4)
α, β, γ (°)	90, 90, 120
*V* (Å^3^)	3533.3 (4)
*Z*	6
Radiation type	Cu *K*α
μ (mm^−1^)	3.42
Crystal size (mm)	0.3 × 0.01 × 0.01
	
Data collection	
Diffractometer	Bruker D8 Venture
Absorption correction	Multi-scan (*SADABS*)
*T*_min_, *T*_max_	0.683, 1.000
No. of measured, independent and observed [*I* > 2σ(*I*)] reflections	32168, 1483, 1250
*R*_int_	0.063
(sin θ/λ)_max_ (Å^−1^)	0.607
	
Refinement	
*R*[*F*^2^ > 2σ(*F*^2^)], *wR*(*F*^2^), *S*	0.040, 0.097, 1.05
No. of reflections	1483
No. of parameters	100
No. of restraints	0
H-atom treatment	Calculated positions, constrained refinement
Δρ_max_, Δρ_min_ (e Å^−3^)	0.33, −0.35
Computer programs: *SADABS* (Bruker, 2002 ▸), *APEX3* (Bruker, 2016 ▸), *SHELXL2018* (Sheldrick, 2015 ▸) and *OLEX2* (Dolomanov *et al.*, 2009 ▸).	
